# TET1 inhibits gastric cancer growth and metastasis by PTEN demethylation and re-expression

**DOI:** 10.18632/oncotarget.8900

**Published:** 2016-04-21

**Authors:** Yao-fei Pei, Ran Tao, Jian-fang Li, Li-ping Su, Bei-qin Yu, Xiong-yan Wu, Min Yan, Qin-long Gu, Zheng-gang Zhu, Bing-ya Liu

**Affiliations:** ^1^ Shanghai Key Laboratory of Gastric Neoplasms, Department of Surgery, Shanghai Institute of Digestive Surgery, Ruijin Hospital, Shanghai Jiao Tong University School of Medicine, Shanghai 200025, PR China; ^2^ Department of Hepatobiliary-Pancreatic Surgery, Zhejiang Province People's Hospital, Hangzhou 310014, PR China

**Keywords:** TET1, 5-methylcytosine, 5-hydroxymethylcytosine, gastric cancer, PTEN

## Abstract

Ten-Eleven Translocation 1 (TET1) is a member of ten eleven translocation enzymes, which convert 5-methylcytosine (5-mC) to 5-hydroxymethylcytosine (5-hmC). TET1 can promote CpG islands demethylation in specific genes and often absent in various cancers. Herein, we found that TET1 expression and 5-hmC content were low in gastric tumors compared to its adjacent non-tumor tissues. Cell proliferation, migration and invasion were enhanced upon TET1 knockdown in gastric cancer cells *in vitro*. This phenomenon was confirmed by an animal xeongraft model. We also found that TET1 directly binds to the promoter region of PTEN and activates its transcription through demethylation of CpG islands. TET1 knockdown activated AKT and FAK pathways, which were suppressed by PTEN. The activation of AKT and FAK facilitated tumor migration, invasion and accelerated cell growth. In conclusion, we found a novel mechanism that TET1 suppresses tumor cell growth, migration and invasion through demethylation of CpG island in PTEN promoter by increasing 5-hmC content. The re-expressed PTEN subsequently down regulates AKT and FAK activity.

## INTRODUCTION

Ten eleven translocation enzymes (TET1, TET2 and TET3) are a family of dioxygenase, which convert 5-methylcytosine (5-mC) to 5-hydroxymethylcytosine (5-hmC) and lead to CpG islands demethylation [1–3]. 5-hmC is regarded as “sixth base” and it is associated with active gene expression while 5-mC stands for gene silence [4]. 5-hmC is always enriched in the promoter region and its content is mainly depended on TET (TET1, TET2 and TET3) levels [5]. The deficiency or mutation of TET proteins and subsequent down-regulation of 5-hmC content is common in various kinds of human cancers. Indeed, TET1 is often absent in breast, hepatic, pancreatic and prostate cancer [6–9]. The reports on the roles of TET1 in human cancers are increasing recently. It has been reported that TET1 was down-regulated in colon tumors from the initial stage and down-regulation of TET1 during colon cancer initiation led to repression of the promoters of WNT pathway inhibitors and resulted in a constitutive activation of the WNT pathway [10].

DNA methylation and demethylation play essential roles in modulating chromosome structure and regulating specific gene expression or repression during cell differentiation. Many diseases are related to aberrant DNA methylation, such as Immunodeficiency–centromeric instability–facial anomalies syndrome (ICF), which is due to DNMT3b inactivation [11, 12]. Similar situations also happened in cancer cells during their initiation, proliferation and metastasis [13, 14]. Gastric cancer is a kind of common cancers, and abnormal DNA methylation correlates with its initiation and progression, especially at the CpG islands located at promoter of tumor repressor genes [15].

Gastric cancer is one of the most common malignant tumors worldwide. Although the improvement in treatment of surgical and chemotherapeutic, the prognosis remains poor because of recurrence and metastasis. TET1 as an epigenetic modulator plays an important role in inhibiting carcinogenesis by regulating 5-hmC and 5-mC content in specific gene promoter. Chuang et al. reported TET1 acts as a protective factor by down regulating 5-mC and up regulating 5-hmC in promoter of invasive-suppressor micro-RNAs in hepatic cancer [7]. Increasing interest has been attracted to TET1 role in carcinogenesis. Though some studies have reported TET1 was down-regulated in gastric cancer, the mechanism how TET1 low expression induces malignant cellular transformation in gastric cancer remains unclear.

In this study, we demonstrated that TET1 expression and 5-hmC content were down-regulated in more than 70% tumor tissues of gastric cancer patients. And TET1 low expression is associated with poor survival. We also revealed that over-expression of TET1 suppressed gastric cancer carcinogenesis through increasing 5-hmC and decreasing 5-mC content at promoter of tumor-suppressor gene PTEN. Knockdown of TET1 induced low expression of PTEN and activation of AKT and FAK pathways, which in turn favored the proliferation, migration and invasion abilities of cancer cells. Our research work firstly clarified that TET1 up-regulates its target gene PTEN through increasing 5-hmC content of its promoter and eventually represses gastric cancer development.

## RESULTS

### TET1 expression is down-regulated in gastric cancer

**Figure 1:**
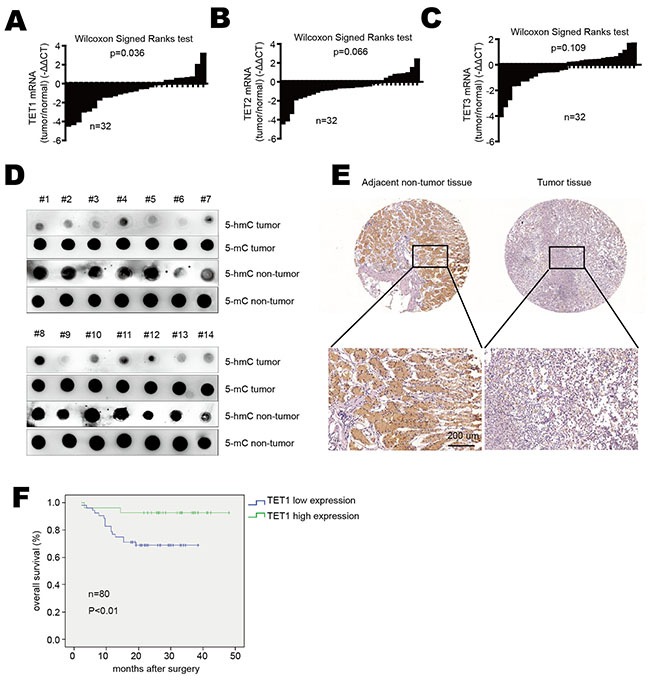
TET1 is down-regulated in gastric cancer **A.** TET1, **B.** TET2 and **C.** TET3 mRNA expression in gastric cancer tissues verse adjacent non-tumor tissues. Data are showed as log_2_ fold changes (tumor/normal). Results were analyzed by the Wilcoxon Signed Ranks test. **D.** 5-hmC and 5-mC content were detected by dot blot in gastric cancer tissues compared to adjacent normal tissues. **E.** IHC analysis of TET1 in gastric cancer tissues and adjacent non-tumor tissues (upper panel 20×, lower panel 200×). **F.** Kaplan–Meier analysis of overall survival in 80 patients with gastric cancer.

In order to explore TET1 expression level in gastric cancer, we analyzed TET1 mRNA expression in tumor tissues and adjacent non-tumor tissues from gastric cancer patients by real-time PCR. We found that TET1 mRNA was significantly reduced in gastric cancer tissues compared with the surrounding non-tumor tissues (p=0.036) (Figure 1A). As TET2 and TET3 are homologues of TET1, we also detected TET2 and TET3 expression in gastric cancer tissues and found that their mRNA levels had no significant difference between tumor tissues and adjacent normal tissues (p=0.066 and p=0.109, respectively) (Figure 1B & 1C). We then used dot blot to detect 5-hmC content in tumor and non-tumor tissues. As shown in Figure 1D, 5-hmC levels were significantly reduced in tumor tissues compared to non-tumor tissues. Immunohistochemical staining (IHC) also showed TET1 down-regulation in tumor tissues (Figure 1E). To confirm these results, we used a tissue array which contains 35 gastric cancer tissues and 6 normal gastric tissues. The IHC analysis showed that TET1 expression was reduced in 28 out of 35 gastric cancer samples compared to non-tumor tissues (Supplementary Figure S1). As TET2 and TET3 expression had no difference between tumor and non-tumor tissues, we focused our research on the roles of TET1 on gastric cancer.

Furthermore, we used another tissue array contains 80 gastric cancer tissues to analyze the correlation between TET1 expression and clinic-pathological parameters. As shown in Table 1, low TET1 expression correlated with local invasion, lymph node metastasis and TNM stage (p<0.05). But no significant correlation was found between TET1 expression and other clinical parameters. The survival analysis also showed patients with low TET1 expression had shorter overall survival than those with high TET1 level (Figure 1F). These results indicate low expression of TET1 in gastric cancer correlates with metastasis and poor prognosis.

**Table 1:** Correlation between TET1 expression level and clinic-pathological parameters

Clinical-pathologic parameters	TET1 expression		p value
	Low (n=53)	High (n=27)	
Age (years)			
≤60	27	12	0.582
≥60	26	15	
Gender			
Male	14	3	0.114
Female	39	24	
Tumor size (cm)			
≤5cm	31	19	0.420
≥5cm	22	8	
Lauren classification			
Intestinal	23	14	0.769
Diffuse	30	13	
Differentiation			
Well, moderately	13	11	0.135
Poorly, undifferentiated	40	16	
Local invasion			
T1, T2	16	17	0.005*
T3, T4	37	10	
Lymph node metastasis			
No	15	19	0.000*
Yes	38	8	
TNM stage			
I, II	25	20	0.022*
III, IV	28	7	

### TET1 suppresses gastric cancer cell proliferation, migration and invasion

To investigate TET1 function in gastric cancer, we knocked down TET1 in NCI-N87 cell by means of two shRNAs (KD1 and KD2), which targeted different sequences of TET1. We also over-expressed TET1 in SGC-7901 (7901-TET1). The efficiency of knockdown and over-expression was confirmed by both qPCR and Western blot. As a result, we observed that 5-hmC content decreased in KD1, KD2 cells while it increased in 7901-TET1cells (Supplementary Figure S2B & S2C).

**Figure 2:**
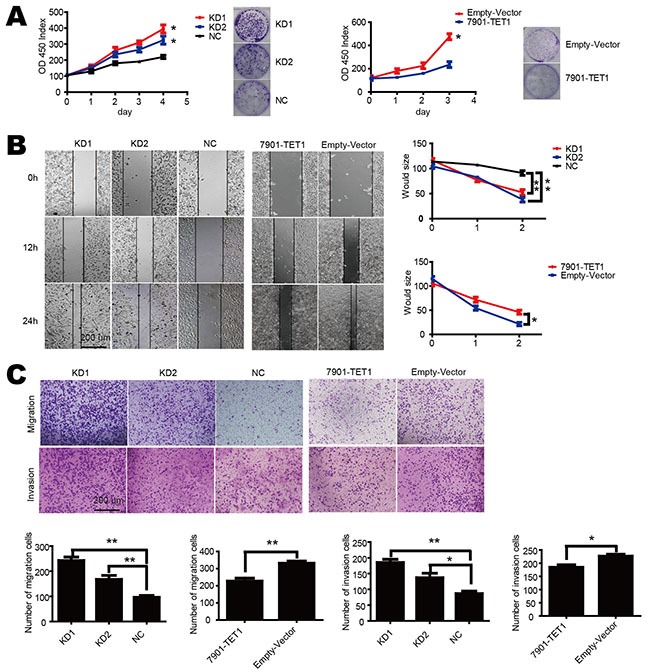
TET1 inhibits cell proliferation, migration and invasion in gastric cell lines **A.** TET1 knockdown accelerates (left) and over-expression decelerates (right) cell proliferation. Cell proliferation was measured by CCK-8. **B.** Wound healing assay of TET1 knockdown in NCI-N87 and TET1 over-expression in SGC-7901. **C.** TET1 knockdown accelerates and over-expression inhibits cell metastasis ability. * p<0.05; ** p<0.01.

To investigate the role of TET1 on cell proliferation, we knocked down TET1 in NCI-N87 cell, which induced an accelerated cell proliferation (Figure 2A left). When TET1 was over-expressed in SGC-7901, cell proliferation was significantly inhibited (p<0.05) (Figure 2A right). Moreover, we performed wound healing assay and transwell assay to investigate the role of TET1 on migration and invasion. In wound healing assay, the distance of cells between wound edges was narrower in KD1 and KD2 than in NC group (p<0.01). In contrast, TET1 over-expression inhibited cell motility and induced broader distance between wound edges in 7901-TET1 as compared to Empty vector group (p<0.05) (Figure 2B). In a migration and invasion assay, we observed similar results. KD1 and KD2 cells had higher migration and invasion rate than NC group (p<0.05). On the contrary, 7901-TET1 showed decreased migration and invasion ability as compared to Empty-vector group (p<0.05) (Figure 2C). These results indicate that TET1 acts as a tumor suppressor, which suppresses gastric cancer cell proliferation, migration and invasion.

### TET1 suppresses tumor progress by up regulating PTEN

**Figure 3:**
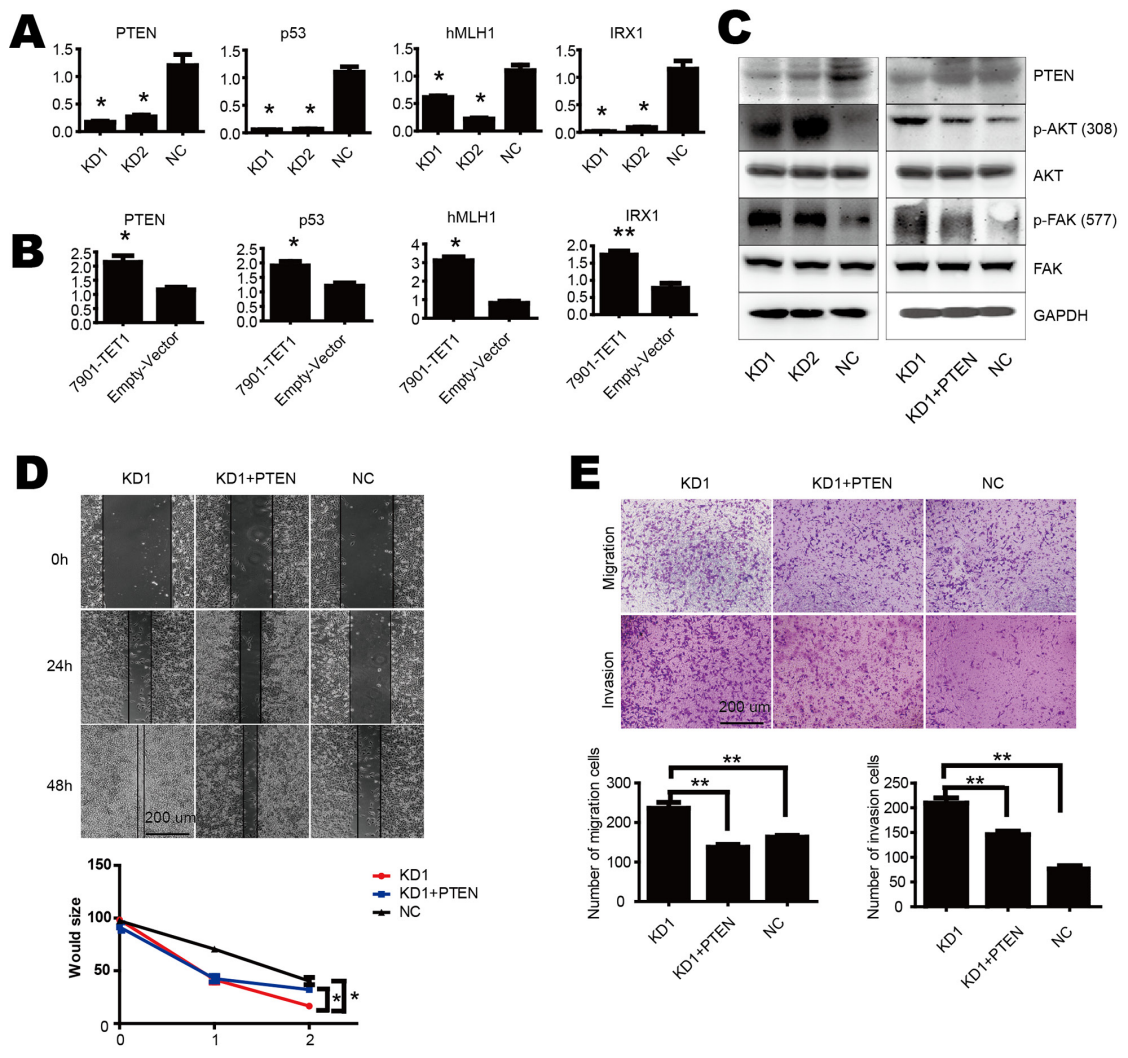
TET1 suppresses tumor progress by up regulating PTEN **A & B.** TET1 increased mRNA levels of PTEN, p53, hMLH1 and IRX1. **C.** TET1 knockdown increased p-AKT and p-FAK (left), while PTEN re-expression decreased p-AKT and p-FAK level (right). **D.** Would healing assay of rescued effect by PTEN re-expression in TET1 KD cells. **E.** PTEN re-expression in TET1 KD cells inhibited cell migration and invasion. * p<0.05; ** p<0.01.

As DNA methylation is essential for gene expression and TET1 alters methylation status of CpG islands, to investigate the mechanism of TET1 suppresses cell proliferation, migration and invasion of gastric cancer cell, we chose four tumor suppressor genes as candidates which were canonically modulated by promoter methylation status i.e. PTEN, p53, hMLH1 and IRX1. We found their mRNA levels were markedly reduced in KD1 as compared to NC cells (Figure 3A), and up-regulated in 7901-TET1 as compared to Empty-Vector control cells (Figure 3B). These results demonstrate that TET1 is important for the expression of canonical methylation-regulated genes in gastric cancer cell such as PTEN.

AKT and FAK pathway are always involved in cell migration and invasion. More importantly, both of them were down stream of PTEN. The activation of AKT and FAK through phosphorylation promotes metastasis. PTEN interacts with AKT and FAK, inhibits their activity through dephosphorylation of them. So we examined AKT and FAK pathways in our study. We found that the levels of phosphorylated AKT and FAK increased in TET1 knockdown NCI-N87 cells (KD1 and KD2), but the total protein of AKT and FAK was unaltered (Figure 3C left panel). To further confirm the role of PTEN in cell migration and invasion, we performed a rescue assay. We over expressed PTEN in TET1 knockdown cell and observed decreased p-AKT and p-FAK (Figure 3C right panel) and inhibition of cell migration and invasion in TET1 knockdown cells (Figure 3D & 3E). Furthermore, we found low expression of TET1 is associated with PTEN loss and more AKT phosphorylation in the tumor specimens. As shown in Figure S3, IHC and Spearman's correlation analysis showed positive correlation between TET1 and PTEN, while negative correlation existed between TET1 and p-AKT. Collectively, TET1 as a tumor suppressor increases PTEN expression and inhibits AKT and FAK, thus limiting the induction of metastasis.

### TET1 activates PTEN transcription mediated by its catalytic domain

**Figure 4:**
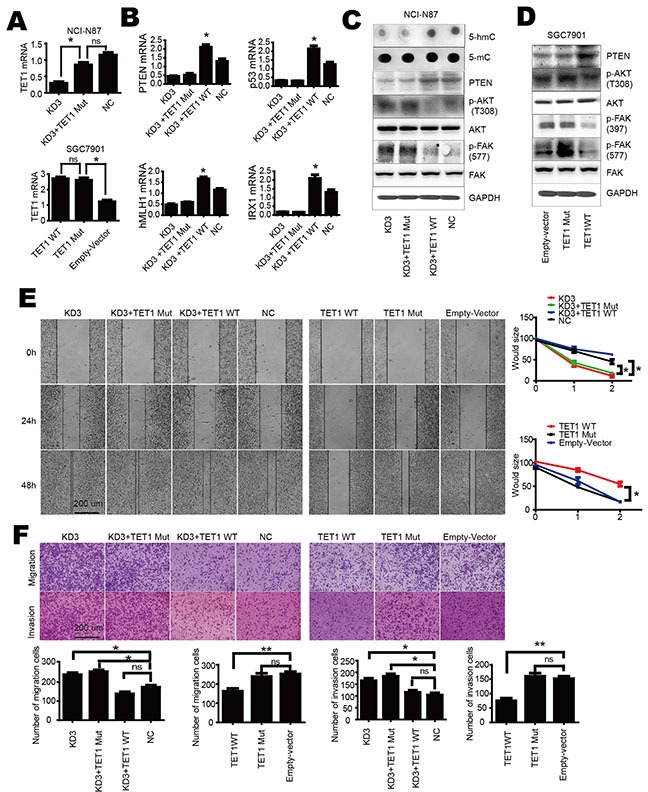
TET1 activates PTEN transcription mediated by its catalytic domain **A.** qPCR analysis of TET1 expression level in KD3 (upper panel) and SGC-7901 (lower panel) cells after over-expression of wild type or mutant TET1. **B.** Over-expression of mutant TET1 has no effect on PTEN, p53, hMLH1 and IRX1. **C.** Over-expression of wild type but not mutant TET1 increases 5-hmC and PTEN level, decreases p-AKT and p-FAK in KD3 cell. **D.** Over-expression of wild type but not mutant TET1 increases PTEN level, decreases p-AKT and p-FAK in SGC-7901 cell. **E & F.** Over-expression of wild type but not mutant TET1 inhibits cell migration and invasion. * p<0.05; ** p<0.01.

TET1 has the ability to convert 5-mC into 5-hmC through its catalytic domain. To investigate the role of TET1 catalytic domain on PTEN and its downstream, we generated a mutated catalytic domain. We mutated amino acids 1672 H to Y and 1674 D to A. In order to investigate the function of TET1 catalytic domain we performed a rescue assay. A shRNA targeting TET1 3′;UTR-shRNA3 was designed to knock down TET1 in NCI-N87 cell (KD3). As wild type and mutant TET1 over-expressed by plasmids lack 3′;UTR, shRNA3 had no effect on the over-expressed wild type and mutant TET1. We used qPCR to confirm transfection efficiency of TET1 mutant and wild type plasmids (Figure 4A). Then we re-expressed wild type TET1 and mutant TET1 in KD3 cells, respectively. As shown in Figure 4B the mutant TET1 had no effect on downstream gene PTEN. Mutant TET1 lost its ability to maintain 5-hmC in genome DNA. Dot blot showed that this wild type but not the mutant TET1 restored 5-hmC content in genomic DNA when plasmids were over expressed in KD3 cells (Figure 4C). Western blot showed that wild type but not mutant TET1 could decrease the phosphorylation of AKT and FAK (Figure 4C & 4D). We also observed that wild type TET1 blocked cell migration, invasion but not mutant TET1 (Figure 4E & 4F). These results show that the catalytic domain is critical for TET1 function in gastric cancer cell and indirectly indicate that 5-hmC content maintained by TET1 catalytic domain is associated with PTEN expression.

### TET1 increases 5-hmC content in the promoter region of PTEN and promotes its demethylation

**Figure 5:**
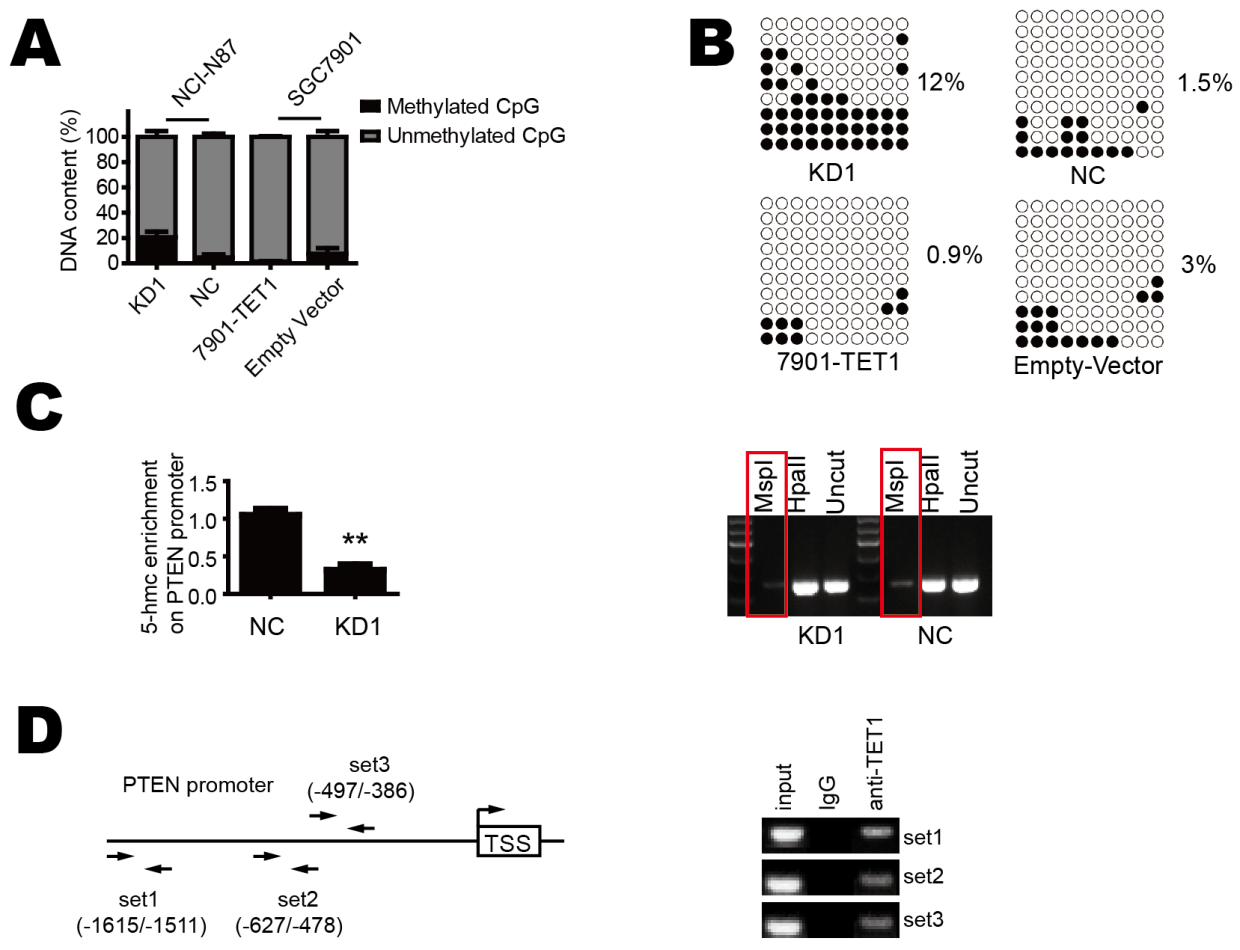
TET1 increases 5-hmC content in the promoter region of PTEN and promotes its demethylation **A & B.** TET1 knockdown in NCI-N87 cell increases PTEN promoter methylation. TET1 over-expression in SGC-7901 cell decreases PTEN promoter methylation. **C.** TET1 knockdown decreases 5-hmC content in PTEN promoter. **D.** The position of ChIP primers and PCR results of ChIP. * p<0.05; ** p<0.01.

We further to explore how TET1 increases PTEN expression. 5hmC has been regarded as a novel demethylation marker associated with carcinogenesis [2, 16, 17]. We hypothesized that TET1 binds to the promoter of PTEN gene and promotes its demethylation by increasing the 5-hmC content in this region. To verify this hypothesis, we used GlucMS-qPCR to detect methylation status of PTEN promoter. We found TET1 knockdown increased methylation in KD1 as compared with NC group. In contrast, when TET1 was over-expressed in SGC-7901, promoter methylation decreased (Figure 5A). The bisulfate sequencing PCR (BSP) also showed similar results (Figure 5B). As TET1 regulation of target genes is mediated by 5-hmC in their promoter, so we used GlucMS-qPCR to detect the 5-hmC content in the promoter region of PTEN and found decreased 5-hmC content in this region in TET1 knockdown cells (Figure 5C left). This phenomenon was also confirmed by Semi-quantitative PCR (Figure 5C right). At last, we performed chromatin immunoprecipitation (ChIP) assay to confirm direct binding of TET1 to PTEN promoter using three sets of primers. ChIP assay showed TET1 does bind to the promoter of PTEN (Figure 5D). These results indicate that TET1 directly binds to the promoter of PTEN, converts 5-mC to 5-hmC, demethylates it and consequently increases PTEN transcription.

### TET1 suppresses gastric cancer cell metastasis and growth *in vivo*

**Figure 6:**
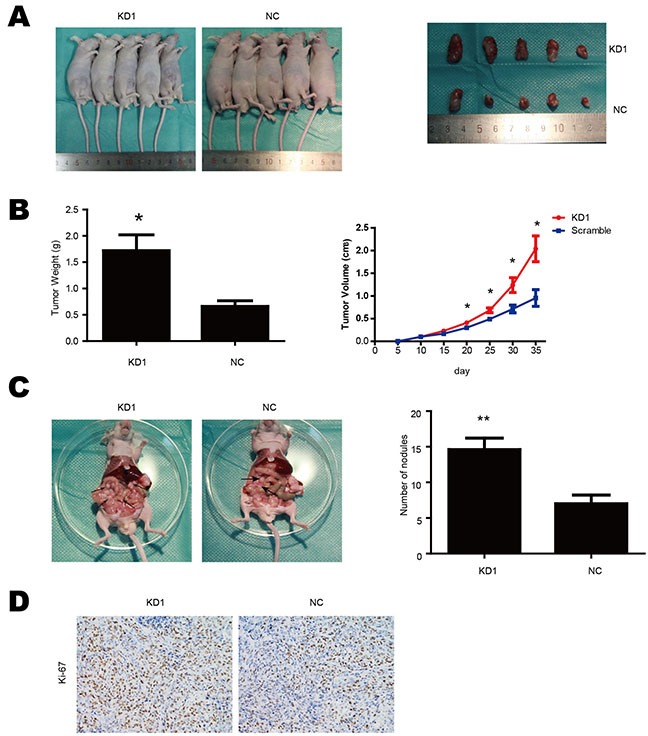
TET1 suppresses gastric cancer cell metastasis and growth in vivo **A.** Subcutaneous tumor formation of KD1 and NC cells. **B.** Tumor growth curves and tumor weight. **C.** TET1 knockdown accelerated tumor peritoneal spreading. **D.** Ki-67 IHC analysis of tumors implanted in nude mice. * p<0.05; ** p<0.01.

Our *in vitro* assay had shown that knockdown of TET1 promoted gastric cancer migration, invasion and proliferation. To investigate TET1 role in gastric cancer cell *in vivo*, we generated a xenograft model. We injected NCI-N87 cells (KD1 and NC) into nude mice subcutaneously and intraperitoneally to observe tumor growth and abdominal metastasis. Consistent with our *in vitro* results, TET1 knockdown was correlated with accelerated tumor growth and increased formation of metastatic peritoneal nodules. The volume and weight of tumor were larger in KD1 group than in the NC control group (Figure 6A & 6B). The KD1 group also had more tumor nodes in the peritoneal cavity (Figure 6C). We also observed that Ki-67 positive cell number was significantly higher in tumors from the KD1 group as compared to the NC group (Figure 6D). These results are consistent with *in vitro* findings and demonstrate that TET1 is a tumor suppressor gene, its knockdown enhances migration, invasion and proliferation of gastric cancer.

### TET1 increases other canonical tumor suppressor genes in gastric cancer cell

**Figure 7:**
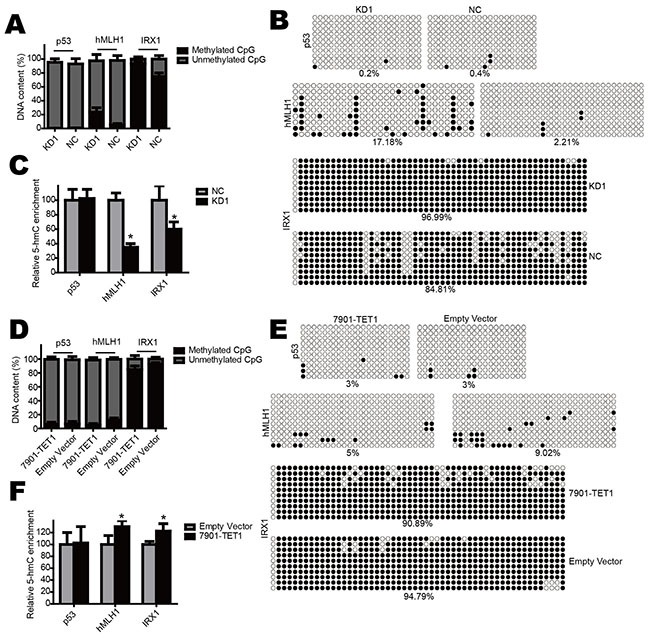
TET1 increases other canonical tumor suppressor genes **A & B.** TET1 knockdown increases promoter methylation of hMLH1 and IRX1 but not p53 in NCI-N87 cell. **C.** TET1 knockdown decreases 5-hmC content in hMLH1 and IRX1 promoter but not p53. **D & E.** TET1 over-expression decreases promoter methylation of hMLH1 and IRX1 but not p53 in SGC-7901 cell. **F.** TET1 over-expression increases 5-hmC content in hMLH1 and IRX1 promoter but not p53.

From qPCR results, we found that the tumor suppressor genes hMLH1, IRX1 and p53 were down-regulated in TET1 knockdown cells. To explore whether these genes were modulated by TET1 through 5-mC and 5-hmC alteration in promoter, we performed GlucMS-qPCR and BSP. The results revealed that when TET1 was knocked down in NCI-N87 cell, the promoter of hMLH1 and IRX1 were significantly methylated (Figure 7A & 7B) and 5-hmC content in such promoter was decreased (Figure 7C). In contrast, when TET1 was over-expressed in SGC-7901 cell, methylation decreased in promoter of hMLH1 and IRX1 (Figure 7D & 7E) while 5-hmC increased (Figure 7F). But TET1 knockdown or over-expression had no effect on p53 promoter. These results indicate that TET1 also increases the expression of hMLH1 and IRX1, which are always modulated by methylation status of their promoter. While the regulation of p53 maybe through other mechanism, which needs further investigation.

## DISCUSSION

5-hmC is a recently discovered epigenetic regulation method and involves in the process of conversion 5-mC to 5-C. Emerging evidence has shown that 5-hmC and TET family might serve unique roles in many biological processes such as gene control mechanisms, DNA methylation, and involved in many diseases, especially cancers. Recent studies have shown that the decrease of 5-hmC may be associated with somatic mutations in IDH, SDH and FH families. But these mutations were rarely observed in gastric cancer [18]. In this study we found TET1 was down-regulated in gastric cancer tissues and cell lines. Clinical parameters showed that gastric cancer patients with low TET1 expression have shorter overall survival than those with high expression of TET1. As the in vitro culture medium used for migration and invasion assay was serum-free, we concluded that TET1 really suppressed gastric cancer cell migration and invasion rather than through inhibiting cell proliferation. Animal experiment also showed TET1 knockdown increases the incidence of metastasis.

PTEN is critical for inhibiting cancer cell migration, invasion and proliferation. Many studies supported that AKT and FAK pathways were excessively activated in tumor cells. The abnormal activation of AKT and FKA promotes cancer progression. PTEN as an inhibitor of AKT and FAK was always down-regulated during tumor development including gastric cancer. Many factors, such as promoter hypermethylation could cause PTEN low expression. A large number of studies have indicated that silencing of PTEN mediated by promoter hypermethylation existed in many kinds of tumors including gastric cancer [19]. In this study, we identified that TET1 knockdown decreased PTEN expression by down-regulating 5-hmC in its promoter.

It has been reported that in hepatic cellular carcinoma, knockdown of TET1 caused CpG islands hypermethylation of invasive-suppressor micro-RNAs which promoted vascular invasion [7]. And in breast cancer, TET1 maintained expression of tissue inhibitors of metalloproteinases, which blocked activity of MMPs, inhibited vascular invasion [8]. F. Neri et al. also revealed that in colorectal cancer cell, TET1 induced expression of DKK3 and DKK4, which inhibited Wnt pathway, eventually inhibited cell growth [10]. In our study, through ChIP assay, we found PTEN expression was modulated by direct binding of TET1 to its promoter. Except PTEN, we also observed down-regulation of p53, hMLH1 and IRX1 mediated by TET1 knockdown. Though TET1 KD decreases 5-hmC content in hMLH1 and IRX1 promoter, whether TET1 directly binds to their promoter needs further validation. And the alteration of p53 expression after TET1 knockdown probably mediated by indirect pathway which needs future investigation.

**Figure 8:**
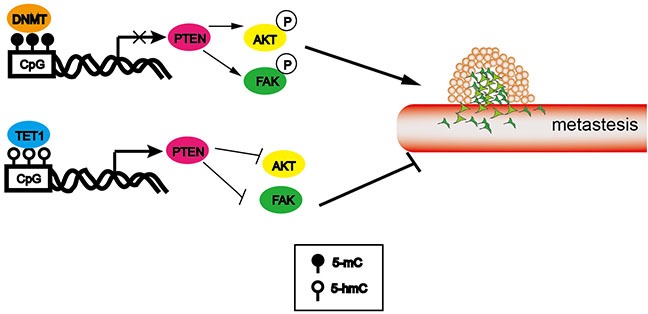
Schematic presentation of TET1 action in metastasis

Collectively, in this study we found PTEN as a novel target gene maintained by TET1 (Figure 8). Though four tumor suppressor genes (PTEN, p53, hMLH1 and IRX1) as candidates were chosen for the mechanism study and three of them were found modulated by promoter 5-hmC content, we didn't know whether TET1 regulates other genes that involved in carcinogenesis. It was a limitation of our study which needed to be investigated in the future.

## MATERIALS AND METHODS

### Gastric cancer specimen and tissue array immunohistochemical staining

32 pairs of tumor tissues and its adjacent non-tumor tissues were collected for RNA extraction and qPCR. Two cohorts of formalin-fixed and paraffin-embedded (FFPE) gastric cancer tissues were also collected. Cohort1 containing 6 normal gastric tissues and 35 tumor tissues was used for IHC. Cohort2 containing 80 gastric cancer tissues was used for survival analysis. All samples were collected from Ruijin Hospital affiliated to Shanghai Jiaotong University School of Medicine. Informed consents were obtained from all patients. The study was approved by the Human Research Ethics Committee of Ruijin Hospital, School of Medicine, Shanghai Jiao Tong University (Permit number: HREC08-028) and carried out in accordance with the guide of Hospital's Protection of Human Subjects Committee. For immunohistochemical staining (IHC), the tissue sections were de-waxed, hydrated and incubated in citrate buffer for antigen retrieval. Endogenous peroxidase activity was inhibited by using 3% H_2_O_2_ then blocked in TBST containing 3% BSA. Tissue sections were incubated with monoclonal anti-TET1 antibody at 4°C over night, washed in TBST 3 times for 10 min and incubated with HRP-conjugated anti-mouse secondary antibody for 2 hr at room temperature, then washed again as per manufacturer's protocol (Dako).

### Cell culture

Gastric cancer cells NCI-N87 and SGC-7901 were purchased from ATCC and cultured in complete RPMI1640 medium containing 10% fetal bovine serum, 100IU/ml penicillin and 100ug/ml streptomycin. In order to generate stable clones, SGC-7901 cells transfected with lentivirus for TET1 over-expression were culture in the medium containing 1ug/ml puromycin for 5 days. The stable clone of NCI-N87 transfected with lentivirus containing TET1 shRNA to knockdown TET1 was selected by flow sorting.

### Constructs

In order to knock down TET1 in gastric cancer cell line, TET1 shRNA was packed in lentivirus using pMD2G, pSPAX2 and shRNA2 according to the protocol provided by Clonetech. The sequences of TET1-shRNA are as follows: N87/KD1: 5′; -CCTTGATAGAATCACTCAGTT-3′;, N87/KD2: 5′;-CCCAGAAGATTTAGAATTGAT-3′;, N87/KD3: 5′;-CCTTGATAGAATCACTCAGTT-3′;, N87/NC: 5′;-CCT AAGGTTAAGTCGCCCTCG-3′;. The full length human TET1 cDNA was obtained from Addgene (Cambridge, MA, USA). Full length human PTEN cDNA was purchased from Shangdong Weizheng Co., Ltd. All the cDNAs were subcloned into pLVX-IRES-Puro vector, then co-transfected into 293T cell with pMD2G, pSPAX2 to pack lentivirus in order to over express TET1 and PTEN in target cells.

### RNA extraction and qPCR analysis

Total RNA was extracted from tissue and gastric cancer cell lines by Trizol Reagent (Invitrogen). Subsequently, RNA was converted to cDNA by reverse transcription kit (Promega, Madison, USA). qPCR was performed using SYBR Green Mix (ABI, CA, USA). The primers designed for detecting target gene expression were as follows: TET1 Forward- CGCTACGAAGCACCTCTCTTA, Reverse- CTTGCATTGGAACCGAATCATTT; TET2 Forward-ATACCCTGTATGAAGGGAAGCC, Reverse- CTTACCCC GAAGTTACsGTCTTTC; TET3 Forward- TCCAGCAACT CCTAGAACTGAG, Reverse- AGGCCGCTTGAATACT GACTG; PTEN Forward- CCCAAGCAACTAGCCCCTC, Reverse- GGCAGCACATCAGGGTAGTC.

### Bisulfate sequencing

Genomic DNA was treated following the guidance of Epi Kit (Qiagen, Germany). The bisulfate-treated DNA samples were then amplified by PCR with primers PTEN Forward: 5′;-GGGGAATCTCTAGGCAAAGGCTGT-3′;, Reverse: 5′;-GTGGTCACCTGGTCCTTTTCACCTG-3′;. P53 Forward: 5′;-GGAGCCCTAGGGCTTGATGGG-3′;, Reverse: 5′;-GGTGCTAAGGAACACAGTGCTTTCAA-3′;, HMLH1 Forward: 5′;-TCTCTTCAGGAGTGAAGGAGGC CA-3′;, Reverse: 5′;- GACTTCCATCTTGCTTCTTTTG GG-3′;; IRX1 Forward: 5′;-GTCCTTCCCGCAGCTGGG-3′;, Reverse: 5′;-AAGAGGAACTAGAAAGGTCC-3′;. PCR products were cloned into T-Easy Vector (Quanshijin, Beijing, China). Ten clones of each treated group were chosen randomly and sent for sequencing and the results were analyzed by online tool (http://quma.cdb.riken.jp/).

### 5-mC and 5-hmC content detection by Gluc MS-qPCR

GlucMS-qPCR to detect 5-mC and 5-hmC content in specific promoter was performed following the protocol of EpiMark 5-hmC and 5-mC Analysis Kit (New England Biolabs). Genomic DNA was treated with T4 Phage b-glucosyltransferase, digested with HpaII and MspI enzyme. HpaII cleaves only a completely unmodified site: any modification (5-mC, 5-hmC or 5-ghmC) at either cytosine blocks cleavage. MspI will recognize and cleave 5-mC and 5-hmC, but not 5-ghmC. The digested products were amplified by PCR. The calculation method of 5-mC and 5-hmC content was described previously [7]. In calculation, samples were normalized to the control group by comparative Ct method. This normalization will give percentage of methylated (HpaII digested samples) and hydroxymethylated (T4-BGT & MspI digested samples). Primers to detect 5-mC and 5-hmC content of specific promoter were as follows. PTEN forward: 5-CTATGTGTTCACGTTCAGCACG-3; reverse: 5-CTAG AGATTCCCCCTTCCCC-3. hMLH1 forward: 5-AACGC TGGGTCCACTCGGGC-3; reverse: 5-GCTTGTGTGC CTCTGCTGAG-3. IRX1 forward: 5-GCGGGAGGGG CGGGAGCGGC-3; reverse: 5-ATCCGCAATCGCGCG CCGAC-3. P53 forward: 5-GTCGCCCGCGAAATCTGA TC-3; reverse: 5-CGGGAGGAGAGGCGAACAG-3.

### DNA extraction and dot blot

Genomic DNA of tissues and cell lines were extracted using DNA isolation kit (Tiangen, Beijin, China). Then genomic DNA was denatured in 0.1M NaOH and treated at 95°C for 10 min, then immediately cooled on ice. Subsequently, genomic DNA was spotted on a nylon membrane. Then the membrane was baked at 80°C for 2 hr, blocked in 5% non-fat milk, incubated with anti-5-hmC and anti-5-mC antibody (GeneTex, USA) at 4°C over night. The membrane was then incubated with HRP-labeled anti-mouse secondary antibody. We detected the signal by ECL kit (Pierce, USA).

### Chromatin immunoprecipitation

Chromatin immunoprecipitation (ChIP) assay was performed in NCI-N87 cell. Cells cultured in 10 cm plates were cross-linked with formaldehyde and the following steps were according to the protocol of ChIP kit (EZ-CHIP catalog#17-371, Millipore). The target DNA fragments were detected by PCR. The primers to detect target DNA fragments are as follows: Set1: forward 5- AAAGGGTTCACCCTAAGCGG-3; reverse 5-CATCGACCTATTCTGCGCCT-3. Set2: forward 5-GGAGCCGGATGAGGTGATAC-3; reverse 5-CGGAT CACAATCGTTCGCAG-3. Set3: forward 5TGCGAAC GATTGTGATCCGA-3; reverse 5-GGTCCCTGCAAG GGGAATAC-3.

### Western blot

Tissues and gastric cancer cells were treated with RIPA containing protease inhibitor cocktail (sigma). The protein samples were kept on ice for 30 min and then centrifuged at 5500g for 30 min at 4°C. Protein concentration was determined by BCA Protein Assay Kit (Thermo Scientific, Rockford, USA). Equivalent amounts of protein samples were loaded in each lane and separated by SDS-PAGE. The protein samples were then transferred on PVDF membrane and blocked with 5% non-fat milk, incubated in primary antibody at 4°C over night. Then the membrane was washed and incubated with HRP-conjugated secondary antibody at room temperature for 2 hours. The membrane was washed again and visualized by ECL method. The TET1, 5-hmC and 5-mC antibodies (cat#GTX300099) were purchased from GeneTex (GeneTex, USA). FAK antibody sampler kit, AKT antibody kit and GAPDH (cat#9330, 9280 and 2118) were from CST (Cell Signaling Technology, USA).

### Cell migration, invasion and proliferation

The ability of cell migration and invasion were determined by transwell assay. Briefly, to determine cell migration potential, RPMI1640 medium containing 10% FBS was added to the lower chamber and 1×10^5^cells suspended in 200ul serum free medium were added to the upper chamber and cultured for 24 hr. For cell invasion, 20ug matrigel was coated on the upper chamber and the following protocols were the same as cell migration, and cultured for 48 hr. Finally, the cells that moved through the membrane were stained with crystal violet and the cell number determined. Cell proliferation was detected by Cell Counting Kit-8 (CCK-8). 0.25 × 10^4^ cells per well were seeded in a 96-well plate and we measured OD450 in 0, 1, 2, 3, 4 day after adding CCK-8.

### Animal model of tumor xenograft

Male BALB/c nude mice were purchased from the Shanghai SLAC Laboratory Animal Co., Ltd (Shanghai, China) and kept in specific pathogen free environment. N87/KD1 cell line in which TET1 had been stably knocked down as well as negative control cells were harvested and suspended in 1×PBS. 1×10^6^ cells were injected into mice subcutaneously and intraperitoneally. At the end of experiment, mice were euthanized. The implanted tumors were stored at −80°C and fixed with 4% paraformaldehyde until usage. All animal experiments were approved by the Laboratory Animal Ethics Committee of Ruijin Hospital (Permit Number: 2013062) and carried out in accordance with the guide of Care and Use Laboratory Animals of Ruijin Hospital, School of Medicine, Shanghai Jiao Tong University.

### Statistics

Data were analyzed by SPSS 19.0 and presented as mean±SD from at least three separate experiments. The Wilcoxon test, Student t test and chi-square test were used in our study. For overall survival, Kaplan–Meier method was used. Differences were considered statistically significant with P < 0.05.

## SUPPLEMENTARY FIGURES



## References

[1] Zhang H, Zhang X, Clark E, Mulcahey M, Huang S, Shi YG (2010). TET1 is a DNA-binding protein that modulates DNA methylation and gene transcription via hydroxylation of 5-methylcytosine. Cell research.

[2] Yang H, Liu Y, Bai F, Zhang JY, Ma SH, Liu J, Xu ZD, Zhu HG, Ling ZQ, Ye D, Guan KL, Xiong Y (2013). Tumor development is associated with decrease of TET gene expression and 5-methylcytosine hydroxylation. Oncogene.

[3] Tahiliani M, Koh KP, Shen Y, Pastor WA, Bandukwala H, Brudno Y, Agarwal S, Iyer LM, Liu DR, Aravind L, Rao A (2009). Conversion of 5-methylcytosine to 5-hydroxymethylcytosine in mammalian DNA by MLL partner TET1. Science (New York, NY).

[4] Lian CG, Xu Y, Ceol C, Wu F, Larson A, Dresser K, Xu W, Tan L, Hu Y, Zhan Q, Lee CW, Hu D, Lian BQ, Kleffel S, Yang Y, Neiswender J (2012). Loss of 5-hydroxymethylcytosine is an epigenetic hallmark of melanoma. Cell.

[5] Williams K, Christensen J, Pedersen MT, Johansen JV, Cloos PA, Rappsilber J, Helin K (2011). TET1 and hydroxymethylcytosine in transcription and DNA methylation fidelity. Nature.

[6] Wu MZ, Chen SF, Nieh S, Benner C, Ger LP, Jan CI, Ma L, Chen CH, Hishida T, Chang HT, Lin YS, Montserrat N, Gascon P, Sancho-Martinez I, Izpisua Belmonte JC (2015). Hypoxia Drives Breast Tumor Malignancy through a TET-TNFalpha-p38-MAPK Signaling Axis. Cancer research.

[7] Chuang KH, Whitney-Miller CL, Chu CY, Zhou Z, Dokus MK, Schmit S, Barry CT (2015). MicroRNA-494 is a master epigenetic regulator of multiple invasion-suppressor microRNAs by targeting ten eleven translocation 1 in invasive human hepatocellular carcinoma tumors. Hepatology (Baltimore, Md).

[8] Hsu CH, Peng KL, Kang ML, Chen YR, Yang YC, Tsai CH, Chu CS, Jeng YM, Chen YT, Lin FM, Huang HD, Lu YY, Teng YC, Lin ST, Lin RK, Tang FM (2012). TET1 suppresses cancer invasion by activating the tissue inhibitors of metalloproteinases. Cell reports.

[9] Sun M, Song CX, Huang H, Frankenberger CA, Sankarasharma D, Gomes S, Chen P, Chen J, Chada KK, He C, Rosner MR HMGA2/TET1/HOXA9 signaling pathway regulates breast cancer growth and metastasis.

[10] Neri F, Dettori D, Incarnato D, Krepelova A, Rapelli S, Maldotti M, Parlato C, Paliogiannis P, Oliviero S (2014). TET1 is a tumour suppressor that inhibits colon cancer growth by derepressing inhibitors of the WNT pathway. Oncogene.

[11] Heyn H, Vidal E, Sayols S, Sanchez-Mut JV, Moran S, Medina I, Sandoval J, Simo-Riudalbas L, Szczesna K, Huertas D, Gatto S, Matarazzo MR, Dopazo J, Esteller M (2012). Whole-genome bisulfite DNA sequencing of a DNMT3B mutant patient. Epigenetics.

[12] Hamidi T, Singh AK, Chen T (2015). Genetic alterations of DNA methylation machinery in human diseases. Epigenomics.

[13] Hoon DS, Spugnardi M, Kuo C, Huang SK, Morton DL, Taback B (2004). Profiling epigenetic inactivation of tumor suppressor genes in tumors and plasma from cutaneous melanoma patients. Oncogene.

[14] Shen L, Kondo Y, Guo Y, Zhang J, Zhang L, Ahmed S, Shu J, Chen X, Waterland RA, Issa JP (2007). Genome-wide profiling of DNA methylation reveals a class of normally methylated CpG island promoters. PLoS genetics.

[15] Tahara T, Arisawa T (2015). DNA methylation as a molecular biomarker in gastric cancer. Epigenomics.

[16] Haffner MC, Chaux A, Meeker AK, Esopi DM, Gerber J, Pellakuru LG, Toubaji A, Argani P, Iacobuzio-Donahue C, Nelson WG, Netto GJ, De Marzo AM, Yegnasubramanian S (2011). Global 5-hydroxymethylcytosine content is significantly reduced in tissue stem/progenitor cell compartments and in human cancers. Oncotarget.

[17] Nestor CE, Ottaviano R, Reddington J, Sproul D, Reinhardt D, Dunican D, Katz E, Dixon JM, Harrison DJ, Meehan RR (2012). Tissue type is a major modifier of the 5-hydroxymethylcytosine content of human genes. Genome research.

[18] Xu W, Yang H, Liu Y, Yang Y, Wang P, Kim SH, Ito S, Yang C, Wang P, Xiao MT, Liu LX, Jiang WQ, Liu J, Zhang JY, Wang B, Frye S (2011). Oncometabolite 2-hydroxyglutarate is a competitive inhibitor of alpha-ketoglutarate-dependent dioxygenases. Cancer cell.

[19] Hino R, Uozaki H, Murakami N, Ushiku T, Shinozaki A, Ishikawa S, Morikawa T, Nakaya T, Sakatani T, Takada K, Fukayama M (2009). Activation of DNA methyltransferase 1 by EBV latent membrane protein 2A leads to promoter hypermethylation of PTEN gene in gastric carcinoma. Cancer research.

