# Supplementary material for: TET1 inhibits gastric cancer growth and metastasis by PTEN demethylation and re-expression
Source: Oncotarget. 2016 Apr 21;7(21):31322–35. doi: 10.18632/oncotarget.8900 (PMC5058759; doi:10.18632/oncotarget.8900)
Supplement: Supplementary file 1 [file oncotarget-07-31322-s001.pdf]

SUPPLEMENTARY FIGURES

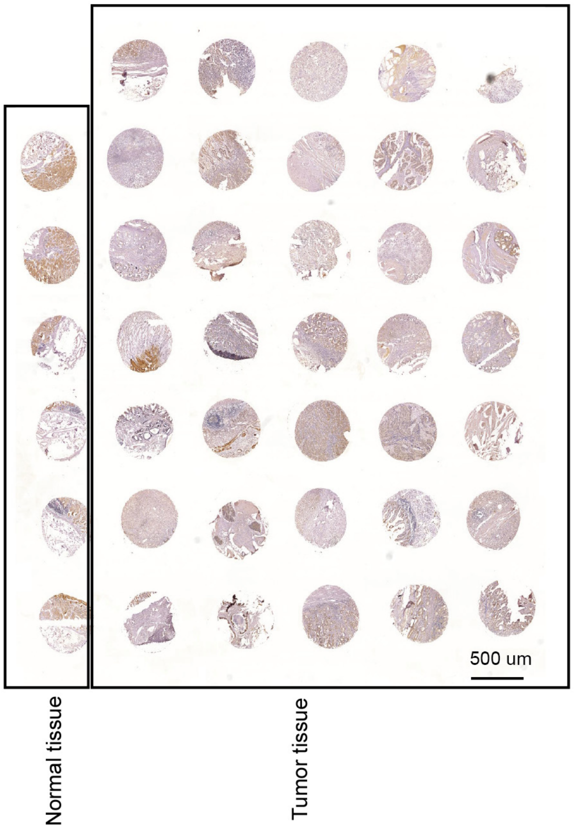

Supplementary Figure S1: IHC analysis of tumor tissues and adjacent non-tumor tissues.

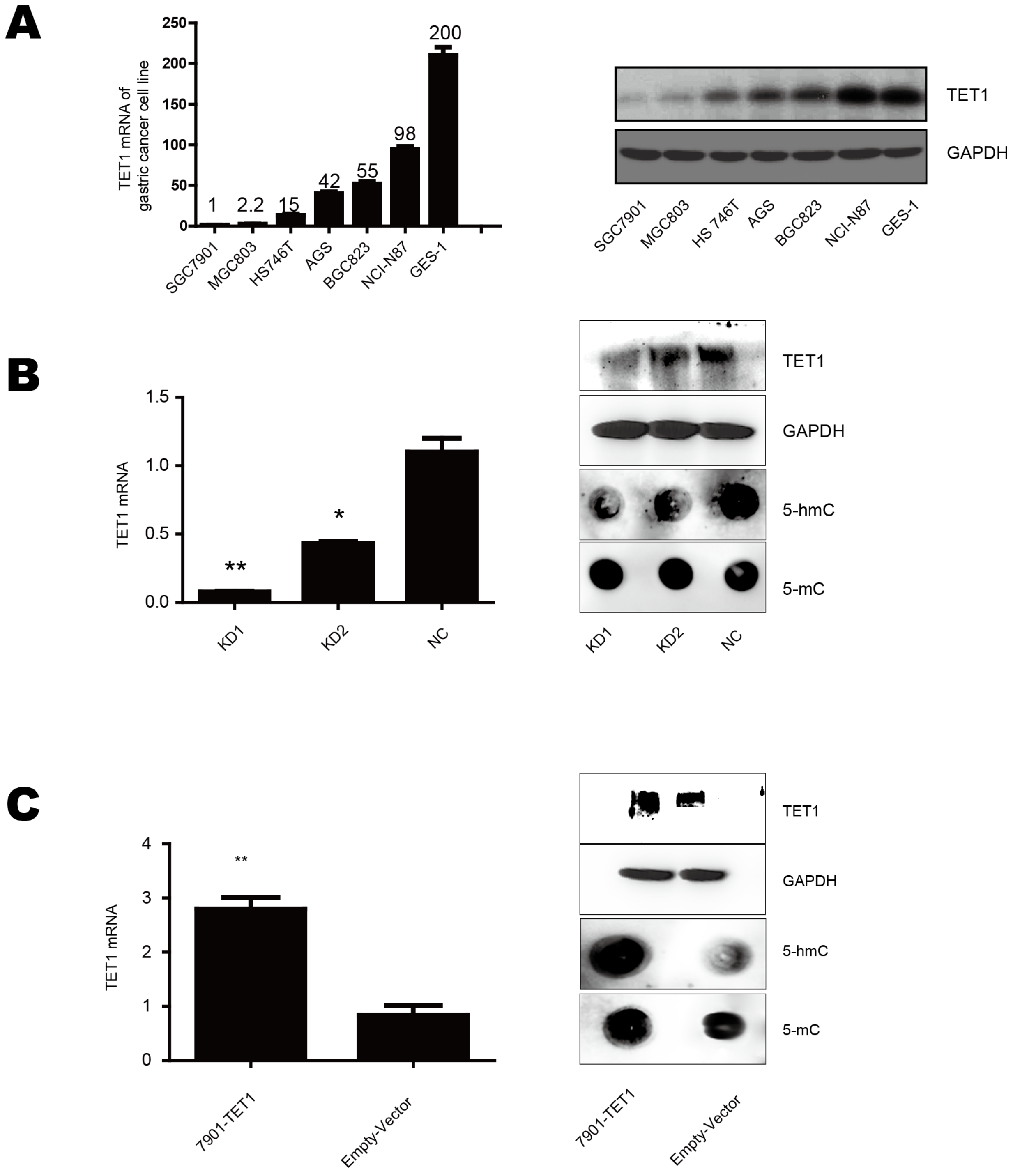

Supplementary Figure S2: A. TET1 level in gastric cancer cell lines. **B & C.** TET1 was knocked down and over-expressed in NCI-N87 and SGC-7901 cells.

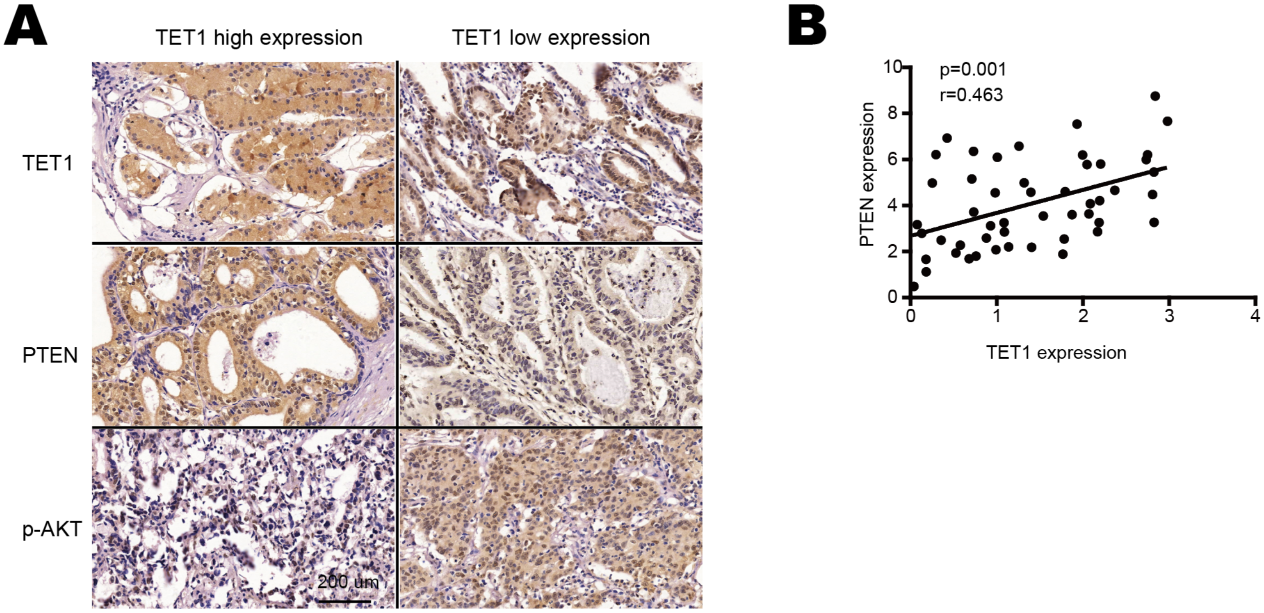

C

	Difference		correlation	
	Chi-square value	p value	R value	p value
TET1 vs PTEN	0.003	0.238	0.59	0.001 *
TET1 vs p-AKT	6.983	0.008	-0.295	0.008*

Supplementary Figure S3: The correlation between TET1, PTEN and p-AKT in specimens. A. IHC analysis showed positive correlation between TET1 and PTEN, while negative correlation existed between TET1 and p-AKT. **B & C.** Spearman's correlation analysis showed positive correlation between TET1 and PTEN, while negative correlation between TET1 and p-AKT.
